# Drinking Waters of London

**Published:** 1858-07

**Authors:** 


Drinking Waters of London.

Dr. Lankester in an excellent article on the *Drinking Waters of the Metropolis*, observes, that

"The water used in London for drinking purposes is obtained from both rivers and springs. The Thames and the New River, and partially other rivers, supply the river water. The spring water is of two kinds. First, from surface wells, obtained by digging through the gravel which covers the London clay in the western parts of the metropolis, and into the clay itself. Secondly, from deep wells, which generally pass through the London clay, and penetrate the chalk below. The surface wells receive the soakage of the water which falls over London, and the water is contaminated by the contents of cesspools, drains, and sewers. The deep wells receive their supply of water from the chalk which forms the sides of the great 'London Basin.' All these waters contain more or less of the following mineral constituents: —1st, carbonate of lime; 2nd, sulphate of lime; 3rd, chloride of sodium; 4th, phosphates and silica; 5th, ammonia; 6th, nitrates and organic matters."

Dr. Lankester concludes that the waters from deep wells as most desirable and unobjectionable as drinking water; that the water from surface wells ought, under no circumstances, to be drunk at all; and that if Thames water is used, it ought to be filtered, or, what is better, boiled and filtered.

